# A Quantity-Dependent Nonlinear Model of Sodium Cromoglycate Suppression on Beta-Conglycinin Transport

**DOI:** 10.3390/ijms25126636

**Published:** 2024-06-17

**Authors:** Ziang Zheng, Junfeng Han, Xinyi Chen, Shugui Zheng

**Affiliations:** 1 College of Information Science and Engineering, Northeastern University, NO. 3-11, Wenhua Road, Shenyang 110819, China; 20215467@stu.neu.edu.cn (Z.Z.);; 2 College of Animal Science and Veterinary Medicine, Shenyang Agricultural University, 120 Dongling Road, Shenyang 110866, China

**Keywords:** quantity-dependent nonlinear model, sodium cromoglycate, suppression, beta-conglycinin, transport

## Abstract

Understanding the transport mechanism is crucial for developing inhibitors that block allergen absorption and transport and prevent allergic reactions. However, the process of how beta-conglycinin, the primary allergen in soybeans, crosses the intestinal mucosal barrier remains unclear. The present study indicated that the transport of beta-conglycinin hydrolysates by IPEC-J2 monolayers occurred in a time- and quantity-dependent manner. The beta-conglycinin hydrolysates were absorbed into the cytoplasm of IPEC-J2 monolayers, while none were detected in the intercellular spaces. Furthermore, inhibitors such as methyl-beta-cyclodextrin (MβCD) and chlorpromazine (CPZ) significantly suppressed the absorption and transport of beta-conglycinin hydrolysates. Of particular interest, sodium cromoglycate (SCG) exhibited a quantity-dependent nonlinear suppression model on the absorption and transport of beta-conglycinin hydrolysates. In conclusion, beta-conglycinin crossed the IPEC-J2 monolayers through a transcellular pathway, involving both clathrin-mediated and caveolae-dependent endocytosis mechanisms. SCG suppressed the absorption and transport of beta-conglycinin hydrolysates by the IPEC-J2 monolayers by a quantity-dependent nonlinear model via clathrin-mediated and caveolae-dependent endocytosis. These findings provide promising targets for both the prevention and treatment of soybean allergies.

## 1. Introduction

Soybean, renowned for its balanced amino acid composition and functional advantages, serves as a highly regarded protein source [1,2]. However, it also poses a risk of dietary allergies in humans and animals [3,4]. Beta-conglycinin, the primary storage protein in soybeans, has been identified as the main soybean allergen. Beta-conglycinin is demonstrated to be a trimeric globulin consisting of three subunits: α’ (72 kDa), α (68 kDa), and β (52 kDa), held together via hydrogen bonding, hydrophobic interaction, and other mechanisms [5]. All three subunits of beta-conglycin were shown to exhibit IgE reactivity in the sera of individuals with a history of soybean allergy and piglets allergic to soybean protein [4,6].

Dietary proteins are primarily broken down by proteolytic enzymes and peptidases within the gastrointestinal tract when ingested by humans or animals. However, a minor proportion of intact allergens or their fragments escape enzymatic hydrolysis, allowing them to be absorbed in the intestine [7,8]. The varying resistance to enzymatic degradation among different dietary proteins strongly correlates with their overall allergenicity [9,10]. The gastrointestinal tract performs dual functions: it digests and absorbs dietary nutrients and simultaneously functions as a protective barrier, exhibiting selective permeability against external factors [11]. The initiation of an immune response against food allergens necessitates the interaction between the allergens and the immune cells present within the intestinal mucosa. Understanding the route and mechanism by which food allergens traverse the intestinal epithelial barrier to access the mucosal immune system beneath the intestinal epithelia is crucial for comprehending the induction of hypersensitivity and developing effective therapeutic approaches. However, the specific mechanism by which beta-conglycinin or its hydrolyzed fragments cross the intestinal mucosal barrier remains to be clarified.

Sodium cromoglycate (SCG), a compound derived from bis-chromone carboxylic acid, actually originates from chromone, a substance separated from the seeds of *Ammi visnaga*, a plant native to Eastern Mediterranean regions. It is noteworthy that the chromone structure exhibits similarities to flavonoids, a class of important bioactive substances. SCG has been proven to be both effective and safe in the prevention of food allergies. Previous studies have demonstrated that SCG suppressed the release of chemical mediators, including histamine from mast cells, thereby stabilizing these cells [12,13]. Nonetheless, the underlying molecular mechanisms by which SCG prevents food allergies, specifically those related to the intestinal absorption and transport of soybean antigens, are still not fully understood.

The objective of the present study was to clarify the pathway and mechanism of beta-conglycinin hydrolysates transported by intestinal epithelial cells, as well as to explore the role of SCG in allergen absorption and transport using the intestinal porcine epithelial cells (IPEC-J2 cells) model.

## 2. Results

### 2.1. Rabbit Anti-Beta-Conglycinin Polyclonal Antibody

The polyclonal antibody against beta-conglycinin exhibited a unique affinity for both beta-conglycinin and its hydrolysates, without cross-reacting with other soybean proteins such as glycinin. This suggested that the antibody possessed excellent binding specificity and was suitable for use in subsequent experimental studies. This polyclonal antibody had been previously utilized in a previous study [14].

### 2.2. Degradation of Soybean Protein under Gastrointestinal Conditions

**Figure 1:**
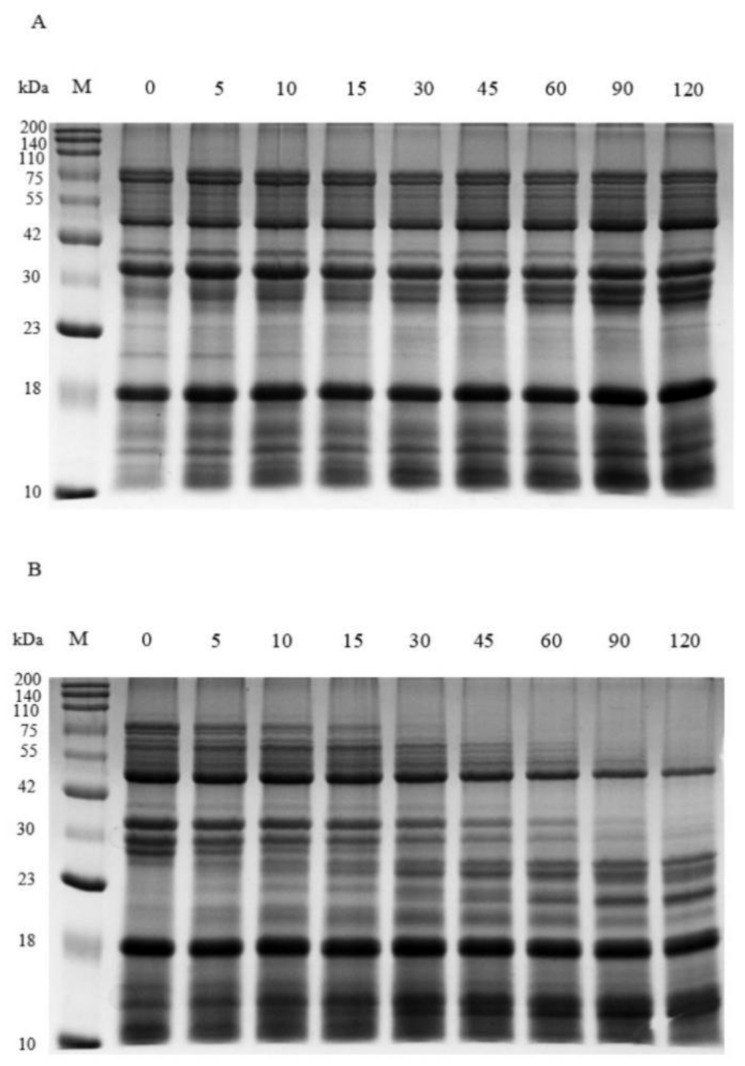
In vitro simulated digestion of soybean meal with simulated salivary fluid (SSF), gastric fluid (SGF), and intestinal fluid (SIF) sequentially. (**A**) Soybean meal was digested in simulated gastric fluid (SGF) for up to 120 min after digestion in simulated salivary fluid (SSF) for 2 min. Samples were collected at various time points (0, 5, 10, 15, 30, 45, 60, 90, and 120 min) and electrophoresed on SDS-PAGE gels. (**B**) The soybean meal was further digested in simulated intestinal fluid (SIF) for up to 120 min following digestion in SGF for 120 min. Samples were taken at different time points (0, 5, 10, 15, 30, 45, 60, 90, and 120 min) and electrophoresed on SDS-PAGE gels. M, molecular weight standard.

Soybean meal was sequentially digested using simulated salivary, gastric, and intestinal fluids, following the physiologically relevant Infogest consensus model. The resulting hydrolysates were then subjected to electrophoresis on SDS-PAGE gels and visualized using Coomassie brilliant blue staining. The results indicated that soybean protein exhibited significant resistance to digestion. Notably, the 52 kDa peptide fragment derived from beta-conglycinin maintained its stability throughout the entire in vitro gastric and intestinal digestion, suggesting that this peptide fragment possessed stronger anti-digestion properties (Figure 1).

### 2.3. Impact of Soybean Meal Hydrolysates, Methyl-Beta-Cyclodextrin (MβCD), Chlorpromazine (CPZ), Amiloride Hydrochloride, and Sodium Cromoglycate (SCG) on the Viability of IPEC-J2 Cells

**Figure 2:**
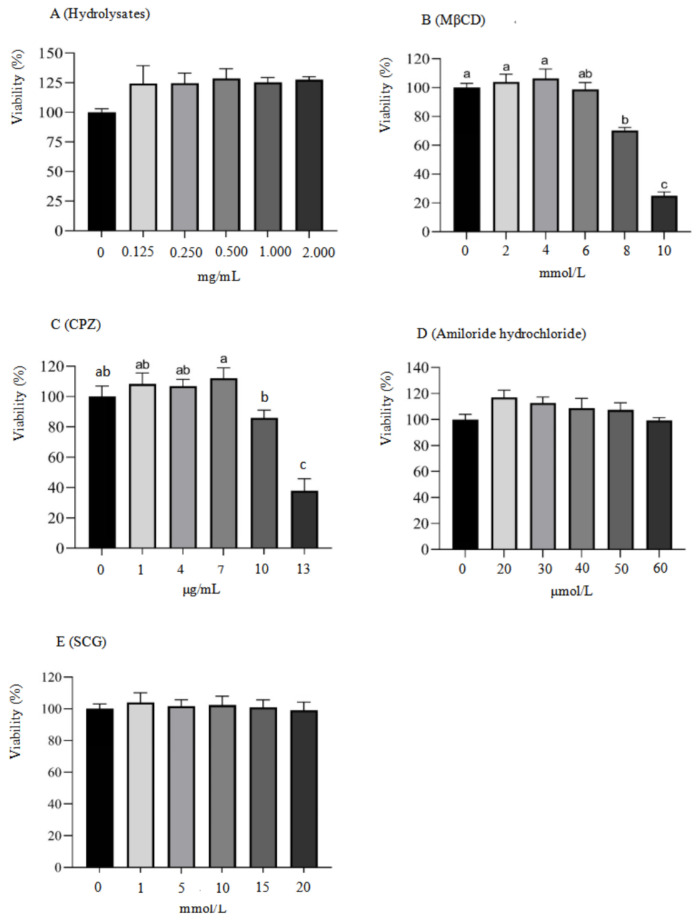
Effect of soybean meal hydrolysates, methyl-beta-cyclodextrin (MβCD), chlorpromazine (CPZ), amiloride hydrochloride, and sodium cromoglycate (SCG) on the viability of IPEC-J2 cells at different concentrations. The IPEC-J2 cells were seeded in 96-well plates and grown to 80–90% confluence. Subsequently, the cells were exposed to soybean meal hydrolysates, MβCD, CPZ, amiloride hydrochloride, and SCG for 24 h at the specified concentrations, respectively. Cell viability was measured with the CCK-8, and the results are expressed as a percentage relative to the control. The data are shown as the mean ± standard deviation (SD), *n* = 3. Different letters are used to indicate statistically significant differences (*p* < 0.05).

The CCK-8 method was employed to assess the effects of soybean meal hydrolysates, methyl-beta-cyclodextrin (MβCD), chlorpromazine (CPZ), amiloride hydrochloride, and sodium cromoglycate (SCG) on the viability of IPEC-J2 cells. The results are presented in Figure 2. The viability of IPEC-J2 cells was not significantly influenced by soybean meal hydrolysates at concentrations ranging from 125 to 2000 μg/mL and SCG at concentrations of 1 to 20 mmol/L (*p* > 0.05). Similarly, the endocytosis inhibitors, including MβCD at concentrations of 2–6 mmol/L, CPZ at concentrations of 1–7 μg/mL, and amiloride hydrochloride at concentrations of 20–60 μmol/L, did not significantly influence the viability of IPEC-J2 cells (*p* > 0.05). However, a significant reduction in IPEC-J2 cell viability was observed when the concentrations of MβCD and CPZ reached 8 mmol/L and 13 μg/mL, respectively, compared to the control group (*p* < 0.05). Therefore, soybean meal hydrolysates at 250 to 2000 μg/mL, SCG at 5, 10, and 20 mmol/L, CPZ at 7 μg/mL, MβCD at 6 mmol/L, and amiloride hydrochloride at 50 μmol/L were selected as the working concentration in this study, respectively.

### 2.4. Transport of Beta-Conglycinin Hydrolysates across IPEC-J2 Cell Monolayers

The IPEC-J2 cells were seeded on Transwell inserts in 12-well plates and cultured to establish compact cell monolayers. The transepithelial electrical resistance (TEER) gradually increased at the beginning and then rose rapidly after day 4. It reached a stable plateau phase on days 8–10, maintaining a TEER value of 1000 Ω·cm^2^. At these times, the cellular transcytosis assay was conducted. Throughout the entire experiment, the TEER was constant and unchanged. Concurrently, the phenol red permeability assay demonstrated a leakage rate of phenol red below 0.5%, suggesting that the allergen did not compromise the integrity of the tight junctions, maintaining their structural integrity.

**Figure 3:**
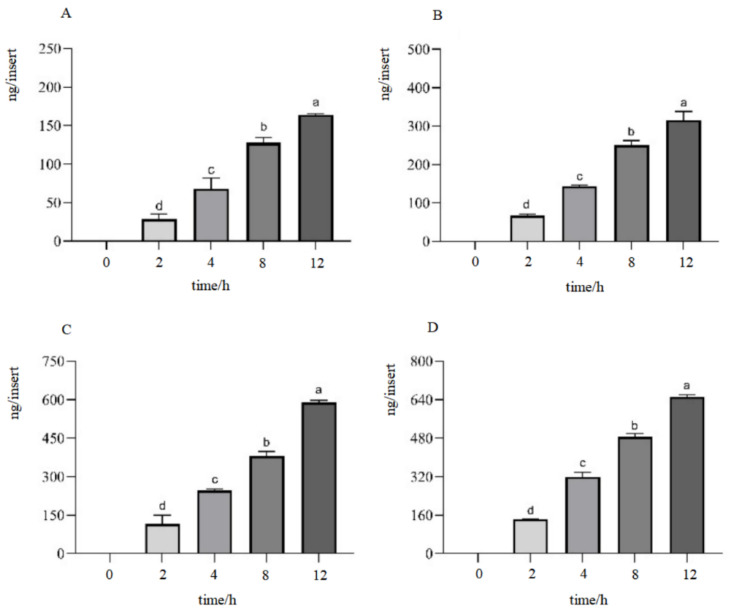
Time-dependent transport of beta-conglycinin hydrolysates through IPEC-J2 cell monolayers. The IPEC-J2 cell monolayers were incubated with beta-conglycinin hydrolysates at concentrations of 0.25 mg/mL (**A**), 0.5 mg/mL (**B**), 1.0 mg/mL (**C**), and 2.0 mg/mL (**D**) for 2 h, 4 h, 8 h, and 12 h, respectively. The contents of beta-conglycinin hydrolysates in basolateral solutions were quantified by ELISA. Data were shown as means ± standard deviation (SD), *n* = 3. Error bars represented the standard deviation of the means. Different letters were used to indicate statistically significant differences (*p* < 0.05).

**Figure 4:**
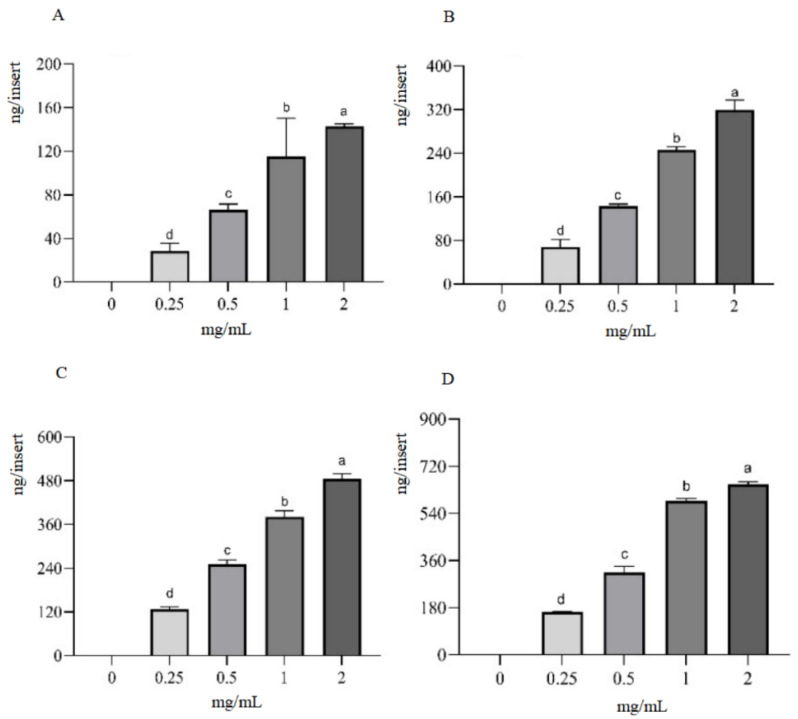
Quantity-dependent transport of beta-conglycinin hydrolysates across IPEC-J2 cell monolayers. The IPEC-J2 cell monolayers were incubated with beta-conglycinin hydrolysates at concentrations varying from 0.25 to 2.0 mg/mL for durations of 2 h (**A**), 4 h (**B**), 8 h (**C**), and 12 h (**D**), respectively. The contents of beta-conglycinin hydrolysates in the basolateral solutions were quantified by ELISA. The results were presented as mean values ± standard deviation (SD), *n* = 3. Error bars represented the standard deviation of the means. Different letters were used to indicate statistically significant differences (*p* < 0.05).

In the time-course study, the IPEC-J2 cell monolayer was exposed to a fixed concentration of hydrolysates for durations of 2 h, 4 h, 8 h, and 12 h. The concentration of beta-conglycinin hydrolysates in the basolateral solutions showed a significant increase with longer incubation times (Figure 3). For the quantity–effect experiment, the IPEC-J2 cell monolayer was incubated with beta-conglycinin hydrolysates ranging from 0.25 to 2.0 mg/mL for a defined time. The concentration of beta-conglycinin hydrolysates in the basolateral solutions significantly increased with higher concentrations in the apical solutions (Figure 4). These observations indicated that the transport of beta-conglycinin hydrolysates across the IPEC-J2 cell monolayers was dependent on both the duration and quantity of exposure.

### 2.5. Identification of Peptide Fragment Absorbed and Transported by IPEC-J2 Cells

**Figure 5:**
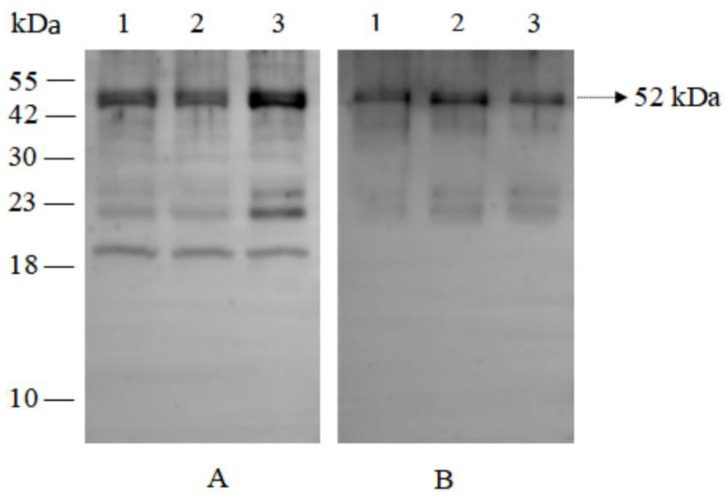
The IPEC-J2 cell monolayers were incubated with beta-conglycinin hydrolysates at concentrations of 0.5 mg/mL (Lane 1), 1.0 mg/mL (Lane 2), and 2.0 mg/mL (Lane 3) for 8 h. Subsequently, Western blot was performed using an antibody against beta-conglycinin to identify peptide fragments absorbed into the cells (**A**) and the peptide fragments present on the basolateral side of the IPEC-J2 cell monolayers (**B**). The results indicated that a 52 kDa peptide fragment derived from beta-conglycinin was absorbed by IPEC-J2 cells and transported across the cell monolayers.

The IPEC-J2 cell monolayers were exposed to soybean meal hydrolysates. Subsequently, the peptide fragments that were absorbed into the IPEC-J2 cells and transported to the basolateral side of the Transwell were detected by Western blot. The results showed that the 52 kDa peptide fragment of beta-conglycinin was capable of being efficiently absorbed by IPEC-J2 cells and transported across the cell monolayer as presented in Figure 5.

### 2.6. Absorption and Transport Pathways of Beta-Conglycinin Hydrolysates

**Figure 6:**
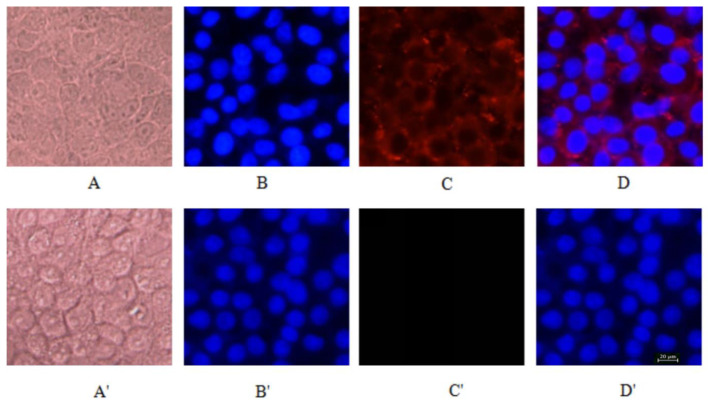
Immunolocalization of beta-conglycinin hydrolysates in IPEC-J2 cell monolayers. The IPEC-J2 cells were seeded onto coverslips placed in a 6-well plate and cultured in DMEM/F12 complete medium for 6 days at 37 °C to form compact cell monolayers. Subsequently, the cells were incubated with DMEM/F12 medium containing 1.0 mg/mL of beta-conglycinin hydrolysates for 8 h. The cells were then fixed using 4% paraformaldehyde and processed following standard immunofluorescence procedures. The prepared sections were observed under a fluorescence microscope. (**A**) Light microscopic image of IPEC-J2 cell monolayers; (**B**) nuclei stained with DAPI; (**C**) immunostaining of beta-conglycinin-derived peptide fragments (red) using a Cy3-labeled goat anti-rabbit secondary antibody following incubation with rabbit anti-beta-conglycinin primary antibodies; (**D**) merged image of (**B**) and (**C**). (**A’**–**D’**) represent the corresponding controls. Scale bar = 20 μm.

**Figure 7:**
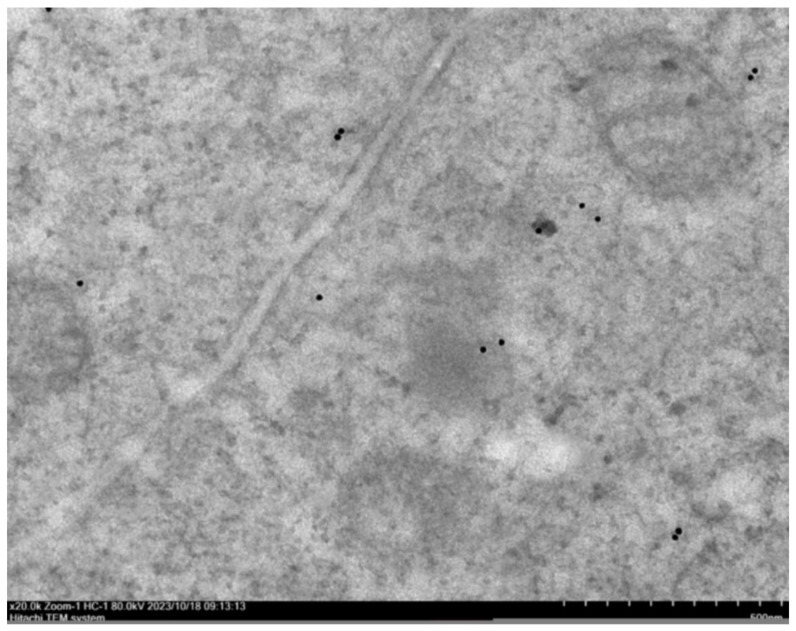
Immunoelectron micrograph of beta-conglycinin hydrolysates absorbed into cell monolayers. The IPEC-J2 cells were cultured in a 6-well plate using DMEM/F12 complete medium for 6 days to establish dense cell monolayers. Following this, the cells were incubated with a DMEM medium containing 1.0 mg/mL of beta-conglycinin hydrolysates for 8 h. After incubation, the cell monolayers were thoroughly washed, fixed, and then processed according to the standard procedure for immunoelectron microscopy. The resulting ultrathin sections were photographed using a transmission electron microscope. The immunogold-labeled beta-conglycinin fragments are presented as black dots in the micrograph.

To investigate the absorption and transport pathways of beta-conglycinin hydrolysates, immunofluorescence and immunoelectron microscopy techniques were utilized to visualize the subcellular distribution of these hydrolysates. As indicated in Figure 6, the nuclei of intestinal epithelial cells were encircled by red fluorescent hydrolysates, suggesting that beta-conglycinin hydrolysates were internalized into the IPEC-J2 cells and distributed throughout the cell cytoplasm. Furthermore, the immunoelectron microscopy showed the presence of beta-conglycinin hydrolysates within the cell cytoplasm, while no beta-conglycinin hydrolysates were detected in the intercellular spaces where the tight junctions remained intact (Figure 7).

### 2.7. Influence of Endocytosis Inhibitors on the Cellular Absorption and Transport of Beta-Conglycinin Hydrolysates

**Figure 8:**
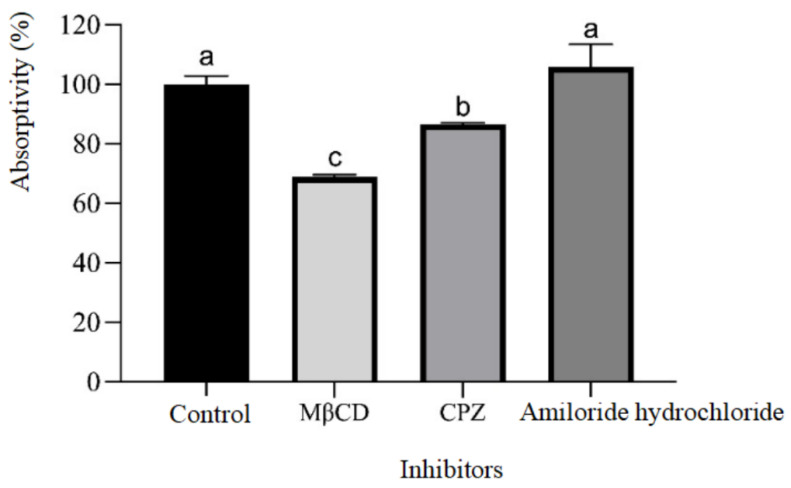
Effects of different endocytosis inhibitors on the absorption and transport of beta-conglycinin hydrolysates by IPEC-J2 cells. The IPEC-J2 cells were seeded in 6-well plates and cultured in DMEM/F12 medium for 6 days at 37 °C in a CO_2_ incubator. The cell monolayers were pretreated with methyl-beta-cyclodextrin (MβCD) (6 mmol/L), chlorpromazine (CPZ) (7 μg/mL), or amiloride hydrochloride (50 μM) in DMEM/F12 basal medium for 30 min, respectively. Subsequently, the cells were cultured in the medium containing the aforementioned concentrations of the respective inhibitors as well as beta-conglycinin hydrolysates (1.0 mg/mL) for 8 h. The intracellular content of beta-conglycinin hydrolysates was determined using ELISA. The absorption rate was expressed as the percentage of beta-conglycinin hydrolysates absorbed by cells with and without the respective inhibitors. Data are presented as means ± standard deviation (SD) with *n* = 3. Error bars represent the standard deviation of the means. Significant differences (*p* < 0.05) are indicated by different letters.

The role of endocytosis inhibitors—MβCD, CPZ, and amiloride hydrochloride—on the cellular absorption and transport of beta-conglycinin hydrolysates was further explored. As presented in Figure 8, the absorption rate of beta-conglycinin hydrolysates by IPEC-J2 cells, when treated with MβCD, CPZ, and amiloride hydrochloride, was 68.84%, 86.54%, and 100%, respectively. Consequently, the suppression rate of the three inhibitors on the cellular absorption and transport of beta-conglycinin hydrolysates was calculated to be 31.16%, 13.46%, and 0%, respectively. These results indicated that the endocytosis inhibitors MβCD and CPZ had significant suppressive effects on the absorption and transport of beta-conglycinin hydrolysates by IPEC-J2 cells.

### 2.8. Effect of SCG on Cellular Absorption and Transport of Beta-Conglycinin Hydrolysates

**Figure 9:**
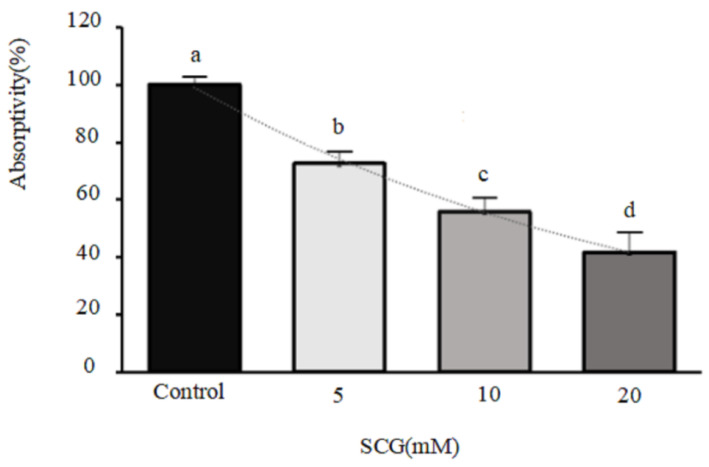
Effects of sodium cromoglycate (SCG) on cellular absorption and transport of beta-conglycinin hydrolysates in IPEC-J2 cells. The IPEC-J2 cells were seeded in 6-well plates and cultured in DMEM/F12 medium for 6 days at 37 °C in a CO_2_ incubator. The cell monolayers were pretreated with 5 mmol/L, 10 mmol/L, and 20 mmol/L of SCG for 30 min, respectively. Following pretreatment, the cells were cultured in a medium containing the aforementioned concentrations of SCG as well as beta-conglycinin hydrolysates (1.0 mg/mL) for 8 h. The intracellular content of beta-conglycinin hydrolysates was determined using ELISA. The absorption rate was calculated by quantifying the proportion of beta-conglycinin hydrolysates absorbed by the cells in the presence and absence of SCG. Data were presented as means ± standard deviation (SD) with *n* = 3. Error bars represent the standard deviation of the means. Significant differences (*p* < 0.05) are indicated by different letters.

The effects of SCG on cellular absorption and transport of beta-conglycinin hydrolysates by IPEC-J2 cells were further examined in the present study. The results indicated that the presence of SCG significantly changed the absorption and transport of beta-conglycinin hydrolysates. When the cells were treated using 5 mM, 10 mM, and 20 mM SCG, the absorption rates of beta-conglycinin hydrolysates were observed to be 72.58%, 55.78%, and 41.65%, respectively. Consequently, the absorption and transport of beta-conglycinin hydrolysates was suppressed by 27.42%, 44.22%, and 58.35% (*p* < 0.05) at these respective concentrations (Figure 9). These results indicated that SCG exerted a suppressive effect on the absorption and transport of beta-conglycinin hydrolysates in IPEC-J2 cells, exhibiting a quantity-dependent nonlinear suppression model.

## 3. Discussion

Beta-conglycinin, a dietary allergen present in soybeans, has been identified as the primary cause of soybean-induced allergies in humans, piglets, and other young animals [15,16]. To acquire a deeper understanding of the sensitization mechanism of beta-conglycinin, we conducted an in vitro digestion of soybean meal in accordance with the infogest consensus model and further investigated the absorption and transport of the hydrolysates by IPEC-J2 cells to elucidate the mechanism by which beta-conglycinin traverses the intestinal mucosal barrier and reaches the subepithelial tissues of the intestine.

Allergenic proteins usually exhibit greater resistance to digestion compared to non-allergenic ones. Proteins that are resistant to gastrointestinal digestion possess a greater opportunity to initiate an immune response, although this attribute does not solely define dietary allergens [17,18]. Some investigations have also shown that although the intact allergenic protein lacks the necessary stability for gastrointestinal digestion, the resulting fragments exhibit resistance to digestion [10]. In this study, we found a significant number of digestion-resistant fragments within the hydrolysates, particularly the peptide fragment with a molecular weight of 52 kD that remained stale even after undergoing simulated salivary digestion for 2 min, gastric digestion for 120 min, and further intestinal digestion for another 120 min under in vitro conditions. Our findings are in line with earlier research [19], indicating that the digestion-resistant peptide fragment possesses the potential to cross the intestinal mucosal barrier and activate immune cells residing within the subepithelial tissue. 

In order to explore the underlying mechanisms of the absorption and transport of beta-conglycinin hydrolysates across the intestinal epithelial barrier, the IPEC-J2 cells, which were derived from the mid-jejunal epithelial cells of neonatal piglets, were used in the present study. The IPEC-J2 cells stand out as a unique model. Unlike other cell lines, it is non-transformed and non-immortalized, retaining its columnar epithelial characteristics [20]. Under optimal in vitro conditions, the IPEC-J2 cells proliferate and form a confluent monolayer. This monolayer expresses tight junction proteins within their apical membrane [21]. Therefore, these cells offer a more authentic representation of small intestinal columnar epithelial cells compared to other cell lines [22].

As we all know, the intestinal mucosal barrier actually consists of a mucus layer, a monolayer of epithelial cells, the lamina propria, and the submucosa. However, IPEC-J2 cells are almost entirely composed of columnar epithelial cells, without other cell types [23]. Therefore, these cells cannot fully simulate the state of the small intestinal mucosa in vivo. In fact, we used IPEC-J2 cells to construct a monolayer model only to explore the role of columnar epithelial cells in allergen absorption and transport, and other factors were not considered in this study.

Macromolecular allergens traverse the intestinal mucosal barrier via two primary routes: the paracellular and transcellular pathways [24]. In the paracellular pathway, macromolecules diffuse through intercellular spaces when the tight junctions between epithelial cells are disrupted. Conversely, the transcellular pathway for macromolecules involves a complex process known as transcytosis. This process includes the endocytosis on the luminal membrane of the epithelia followed by the exocytosis on the basement membrane [25,26].

In this study, we conducted a cellular transcytosis assay using IPEC-J2 cell monolayers cultured on porous support membranes that separated the apical and basal compartments. After reaching its peak value, the transepithelial electrical resistance (TEER) remained constant throughout the transcytosis experiment, indicating that the intercellular tight junctions in the IPEC-J2 monolayer maintained their integrity. Phenol red, a water-soluble small molecule, is poorly absorbed by intestinal epithelial cells and difficult to transport via the transcellular pathway, thus serving as a reliable indicator for paracellular leakage [27]. This research demonstrated that phenol red exhibited extremely low leakage rates, further confirming the intactness of the tight junctions within the IPEC-J2 cell monolayers. In this study, the cellular transcytosis assay showed that the transport of beta-conglycinin hydrolysates across the IPEC-J2 cell monolayer occurred in a manner that was dependent on both time and quantity. Furthermore, it was found that the 52 kDa peptide fragments originating from beta-conglycinin were absorbed and transported by the small intestinal epithelial cells. The cellular absorption and transport assay was conducted to investigate the absorption and transport pathway of peptide fragments in small intestinal epithelial cells of piglets. Following the incubation of IPEC-J2 cells with beta-conglycinin hydrolysates, beta-conglycinin-specific antibodies were utilized to locate the position of beta-conglycinin hydrolysates within the IPEC-J2 cell monolayers by both immunofluorescence and immunoelectron microscopy. The results indicated that beta-conglycinin hydrolysates were present within the IPEC-J2 cells. The tight junctions connecting the epithelial cells maintained their structural integrity, and no beta-conglycinin hydrolysates were observed to traverse the epithelial intercellular spaces. Based on these results, it can be concluded that the beta-conglycinin hydrolysates traversed the IPEC-J2 cell monolayer through the transcellular pathway. This finding was in line with the previous studies that demonstrated the endocytosis-mediated transport of proteins including beta-lactoglobulin and HRP through intestinal epithelial cells, both in vivo and in vitro [28,29,30].

Endocytosis inhibitors, such as MβCD, CPZ and amiloride hydrochloride are frequently used in the investigation of macromolecule endocytosis mechanisms. As a heptasaccharide with a hydrophobic core, MβCD exhibits a strong affinity for cholesterol. By extracting cholesterol from cell membranes, it suppresses both caveolae-dependent and clathrin-dependent endocytosis [31,32,33]. CPZ, a cationic amphipathic drug, is capable of disrupting the assembly and disassembly of clathrin lattices on cell surfaces, thereby hindering clathrin-mediated endocytosis [34]. This inhibitor has also been observed to relocate clathrin and adaptor protein complex-2 from the plasma membrane to the endosomal membrane [35]. Amiloride hydrochloride, however, is capable of lowering submembranous pH and blocking Rac1 and Cdc42 signaling, thereby suppressing macropinocytosis [36]. In the current study, the results showed that the inhibitors MβCD and CPZ effectively reduced the absorption and transport of beta-conglycinin hydrolysates by IPEC-J2 cells. Conversely, amiloride hydrochloride did not influence the absorption and transport process. The suppression rates for MβCD, CPZ, and amiloride hydrochloride on the cellular absorption and transport of beta-conglycinin hydrolysates were 31.16%, 13.46%, and 0%, respectively. These observations indicated that the mechanism of beta-conglycinin hydrolysates absorption into IPEC-J2 cells involved both clathrin- and caveolae-mediated endocytosis, rather than macropinocytosis.

SCG, as an anti-allergic medication, has been reported to effectively suppress the release of 5-hydroxytryptamine, histamine, and other mediators involved in allergic reactions. Consequently, it prevents these mediators from exerting their adverse effects on tissues. Previous studies have demonstrated that SCG is capable of influencing the intestinal permeability of molecules ranging in size, including mannitol and lactulose [37,38]. In a clinical study, it was found that the oral administration of a certain dosage of SCG successfully prevents allergic reactions to soybeans [39]. In the present study, it was observed that SCG effectively suppressed the absorption and transport of beta-conglycinin hydrolysates by IPEC-J2 cells by a quantity-dependent nonlinear model. This result was consistent with an earlier research that demonstrated that SCG was capable of suppressing the absorption and transport of allergen Gly m Bd 30K through clathrin- and caveolae-dependent endocytosis [40]. Therefore, based on a comprehensive analysis of the results of this study, we proposed that SCG suppressed the absorption and transport of beta-conglycinin via clathrin- and caveolae-dependent endocytosis, and this suppression constituted a crucial mechanism in its ability to prevent soybean allergies.

## 4. Methods and Materials

### 4.1. Preparation of Rabbit Anti-Beta-Conglycinin Polyclonal Antibody

The purification of beta-conglycinin from soybeans was carried out using the isoelectric point precipitation method following a previously reported protocol [9]. Briefly, defatted soybean flour was extracted with Tris-HCl buffer (pH 8.0). After centrifugation, the supernatant was adjusted to pH 6.4 and stored overnight at 4 °C. Following another centrifugation, the supernatant was adjusted to pH 5.5, stirred for 30 min, and centrifuged again. Then, the supernatant was further adjusted to pH 4.8 and centrifuged. The precipitate obtained primarily contained crude beta-conglycinin. This crude fraction was loaded onto a Sepharose CL-6B column and eluted with phosphate buffer (pH 7.6). Fractions containing beta-conglycinin were collected, lyophilized, and stored in aliquots at −80 °C.

Subsequently, the rabbit anti-beta-conglycinin polyclonal antibody was prepared in accordance with a previously established method [14]. Briefly, the purified beta-conglycinin was dissolved in sterile PBS and emulsified with complete adjuvant. The vaccine was injected intradermally at multiple sites on the rabbit’s back. Boosters were prepared similarly but with Freund’s incomplete adjuvant, administered every two weeks, for a total of seven boosters. Rabbit blood was collected from the anterior vena cava, and the sera were separated and stored at −80 °C for future use.

### 4.2. Simulated Digestion In Vitro

The digestion of soybean meal (COFCO Corporation, Beijing, China) in vitro was sequentially conducted using simulated salivary fluid (SSF) containing 15.10 mM KCl, 3.70 mM KH_2_PO_4_, 13.60 mM NaHCO_3_, 0.15 mM MgCl_2_, 1.50 mM CaCl_2_, and 0.06 mM (NH_4_)_2_CO_3_ (pH 7.0); gastric fluid (SGF) containing 6.90 mM KCl, 0.90 mM KH_2_PO_4_, 25.00 mM NaHCO_3_, 47.20 mM NaCl, 0.12 mM MgCl_2_, 0.15 mM CaCl_2_, and 0.50 mM (NH_4_)_2_CO_3_ (pH 3.0); and intestinal fluid (SIF) containing 6.80 mM KCl, 0.80 mM KH_2_PO_4_, 85.00 mM NaHCO_3_, 38.40 mM NaCl, 0.33 mM MgCl_2_, and 0.60 mM CaCl_2_ (pH 7.0) in accordance with the COST action Infogest consensus protocol [41]. Initially, 600 milligrams of soybean meal was suspended in 400 microliters of distilled water and blended with 1000 microliters of SSF. This mixture was then agitated and incubated at 37 °C for 2 min to simulate oral digestion. Subsequently, for gastric digestion, 2000 microliters of SGF and pepsin (P7012, Sigma-Aldrich, St. Louis, MO, USA) were added to the oral digesta. The pH was adjusted to 3 using 5 mol/L HCl while maintaining a pepsin activity of 2000 U/mL. The mixture was agitated and incubated at a temperature of 37 °C for a duration of 120 min. Samples were collected at various time points (0, 5, 10, 15, 30, 45, 60, 90, and 120 min) for SDS-PAGE. Following this, the reaction was stopped by adjusting pH to 7.0 using 5 mol/L NaOH. Next, the gastric digesta was blended with 4000 microliters of SIF containing pancreatin (P7545, Sigma-Aldrich, St. Louis, MO, USA) with a trypsin activity of 100 U/mL. The mixture was agitated and incubated at a temperature of 37 °C for 120 min. Samples were taken at different time points (0, 5, 10, 15, 30, 45, 60, 90, and 120 min) for SDS-PAGE. Finally, the reaction was stopped using BBI (T9777, Sigma-Aldrich, St. Louis, MO, USA), and the resulting soybean meal hydrolysates were stored at −80 °C. The concentration of beta-conglycinin hydrolysates in soybean meal hydrolysates was measured by ELISA using the rabbit anti-beta-conglycinin polyclonal antibody.

### 4.3. SDS-Polyacrylamide Gel Electrophoresis (SDS-PAGE)

SDS-polyacrylamide gel electrophoresis (SDS-PAGE) was carried out using a slab gel containing a 5% stacking gel and a 12% resolving gel. Briefly, soybean meal hydrolysates were centrifuged (10,000× *g*, 4 °C, 5 min), and the supernatants were dissolved in a 4×loading buffer and subjected to a boiling water bath for a duration of 10 min. A ten-microliter aliquot of the sample solution (15 μg) was applied onto the gel and resolved using SDS-PAGE. After that, the gels were stained with Coomassie Brilliant Blue (CBB) for the visualization of protein bands.

### 4.4. Cell Culture

The IPEC-J2 cells were cultured in a sterile, humidified environment maintained at a temperature of 37 °C with 5% CO_2_. The cells were seeded in cell culture flasks using Dulbecco’s Modified Eagle Medium/Ham’s F-12 (1:1) medium (Gibco, CA, USA) that was supplemented with 10% fetal bovine serum (FBS), 100 IU/mL penicillin, 100 μg/mL streptomycin, and 0.25 μg/mL amphotericin B.

### 4.5. Cell Viability Assay

Cell viability was measured utilizing a Cell Counting Kit-8 (CCK-8) following the manufacturer’s instructions. In brief, the IPEC-J2 cells were seeded into a 96-well plate at a concentration of 1 × 10^5^ cells per milliliter (1 × 10^4^ cells per well) and incubated using a complete medium composed of DMEM/F12 until reaching 80–90% confluence. Soybean meal hydrolysates, methyl-beta-cyclodextrin (MβCD), chlorpromazine (CPZ), and amiloride hydrochloride were individually dissolved in DMEM basal medium and subsequently diluted to create a series of concentration gradients. The cells were subsequently incubated with these gradient solutions at their respective concentrations for 24 h. Next, the cells were rinsed using PBS and incubated with 10 μL CCK8 for a duration of 1 h. The absorbance at 450 nm was detected utilizing a microplate reader (BioTek, VT, USA). Cell viability was calculated as a percentage using the following formula: cell viability = (OD_T_ − OD_B_)/(OD_C_ − OD_B_) × 100%, where OD_T_ represents the OD value of the treatment wells, OD_C_ refers to the OD value of control wells, and OD_B_ indicates the OD value of the medium without IPEC-J2 cells.

### 4.6. Cellular Transcytosis Assay

The IPEC-J2 cells were seeded onto Transwell inserts (polyethylene terephthalate membrane with a pore size of 0.4 μm, LABSELECT, Beijing, China) in 12-well plates, with each insert containing a density of 1 × 10^5^ cells. In total, 1.5 mL and 0.5 mL of DMEM/F12 medium were added to the basolateral and apical compartments, respectively. The medium was refreshed every other day. The morphological transformation and growth status of the IPEC-J2 cells during the cell culture process were monitored using an inverted microscope. The transepithelial electrical resistance (TEER) was determined using an epithelial resistance/voltage meter (Beijing Jingong Hongtai Technology Co., Ltd., Beijing, China).

The integrity of the IPEC-J2 cell monolayers was evaluated using the marker phenol red in accordance with a previously established protocol [27]. In brief, 0.5 mL of Hank’s balanced salt solution (HBSS) consisting of 42 μM of phenol red was administered to the apical compartment, while 1.5 mL of HBSS was added to the basolateral compartment. After incubating for 1 h at 37 °C, the concentrations of phenol red in both the apical and basolateral solutions were determined at a wavelength of 560 nm after adjusting pH to 10 using 1 N NaOH.

When the trans-epithelial electrical resistance (TEER) arrived at its peak value (1000 Ω·cm^2^) and the phenol red leakage rate remained below 0.5%, the IPEC-J2 cell monolayers were used for the cellular transcytosis assay. Various concentrations of beta-conglycinin hydrolysates (0.25, 0.5, 1.0, and 2.0 mg/mL) were then administered to the apical compartment and incubated for durations of 2 h, 4 h, 8 h, and 12 h accordingly. At the end of each incubation period, the samples from the basolateral compartment were collected for further analysis using ELISA.

Beta-conglycinin hydrolysates at concentrations of 0.5 mg/mL, 1.0 mg/mL, and 2.0 mg/mL were added to the apical compartment and incubated for a duration of 8 h. Subsequently, samples from the basolateral compartment were collected for Western blot. After this, the cell monolayers were thoroughly washed six times with ice-cold HBSS on a shaker, with each wash lasting 10 min. A total of 50 μL of radio immunoprecipitation assay buffer (RIPA buffer) (Beijing Dingguo Changsheng Biotechnology Co., Ltd., Beijing, China) was then added to the Transwell, and the IPEC-J2 cells were gently removed. After being lysed on ice for 30 min, the mixture was centrifuged, and the resulting supernatants were stored at −80 °C for Western blot. Furthermore, throughout the entire cellular transcytosis assay, the TEER and phenol red leakage rate in the IPEC-J2 monolayers were monitored to guarantee the monolayer’s integrity and functionality throughout the process.

### 4.7. Competitive ELISA

The competitive ELISA was employed to determine the levels of beta-conglycinin hydrolysates in the samples from the cellular transcytosis assay following a previously described method [42]. Briefly, 96-well microtiter plates were coated with 200 μL of purified beta-conglycinin (1.0 μg/mL) dissolved in 50 mM bicarbonate buffer (pH 9.6) and incubated overnight at 4 °C. Following thorough washing using phosphate-buffered saline with 0.05% ween 20 (PBST), the plates were subsequently blocked using 1% bovine serum albumin (BSA) at 37 °C for 2 h to prevent nonspecific binding. After three washes with PBST, purified beta-conglycinin was dissolved in PBS and serially diluted to concentrations ranging from 1000 to 0.01 μg/mL (1000, 100, 10, 1, 0.1, and 0.01 μg/mL) to establish a standard curve. Next, 100-μL aliquots of the standard beta-conglycinin solutions or the samples were added to the wells of the beta-conglycinin-coated plates. Afterward, 100 μL of rabbit polyclonal antibody against beta-conlycinin, diluted to a ratio of 1:100,000, was added to each well, and the plates were then incubated at a temperature of 37 °C for 1 h to allow for antigen–antibody binding. Following three washes with PBST, a volume of 100 μL of goat anti-rabbit IgG conjugated with HRP (AS014, ABclonal, Wuhan, China), diluted to a ratio of 1:5000, was added to each well. Subsequently, the plates were incubated for 30 min at 37 °C. Following washing the wells three times with PBST, 100 μL of TMB peroxidase substrate (Solarbio, Beijing, China) was added to each well and allowed to incubate at 37 °C for 10 min. Afterward, the reaction was stopped by adding sulphuric acid (H_2_SO_4_). Finally, the absorbance was determined at a wavelength of 450 nm utilizing a microplate reader (BioTek, VT, USA).

### 4.8. Western Blot

Ten microliter aliquots of the samples (1.5 μg/μL) were applied to the gels and separated by SDS-PAGE. The protein bands present on the gel were then electrophoretically transferred onto a PVDF membrane (BS-PVDF-45-S, Biosharp, Guangzhou, China). The membranes were blocked and incubated overnight at 4 °C with 1:3000-diluted rabbit polyclonal antibodies against beta-conglycinin. After being washed three times with TBST, the membrane was then incubated with 1:5000-diluted goat anti-rabbit IgG antibody conjugated with HRP (AS014, ABclonal, Wuhan, China) for 1 h at 37 °C. Following three washes, the membrane was visualized using Omni-ECL™Pico Light Chemiluminescence Kit (SQ202, EpiZyme, Shanghai, China)

### 4.9. Cellular Absorption and Transport Assay

The IPEC-J2 cells were seeded on the round glass coverslips placed in a 6-well plate at a density of 2.5 × 10^5^ cells/well and maintained in DMEM/F12 complete medium for a duration of 6 days at 37 °C in a CO_2_ incubator to guarantee complete confluence and the formation of a monolayer. Afterward, the cells were treated with DMEM/F12 basal medium containing a concentration of 1.0 mg/mL of hydrolysates for 8 h. The cells were washed six times and fixed with 4% paraformaldehyde for further analysis with immunofluorescence.

Separately, the identical IPEC-J2 cells were seeded in a 6-well plate at a density of 2.5 × 10^5^ cells/well using DMEM/F12 complete medium under the same conditions, and then cultured for 6 days until they reached full confluence and formed a monolayer. These cells were also treated with DMEM basal medium containing 1.0 mg/mL of hydrolysates. After an eight-hour incubation, the cell monolayers were thoroughly washed six times using ice-cold PBS on a shaker. Subsequently, the cells were carefully detached from the plate with a cell scraper, taking care not to disperse the cells. Following centrifugation at 1000 rpm for 2 min, a specialized fixative for immunoelectron microscopy (IEM) (Servicebio, Wuhan, China) was added to the cell pellet. The fixed cells were stored at 4 °C for further analysis using immunoelectron microscopy (IEM).

### 4.10. Immunofluorescence Assay

The cells fixed with 4% paraformaldehyde were washed three times using PBS. After being blocked with 5% goat sera for 1 h at room temperature, the cells were incubated overnight at 4 °C with rabbit anti-beta-conglycinin antibody. Following another three washes using PBS, the cells were incubated with Cy3-labeled goat anti-rabbit antibody for 50 min at room temperature. After three more washes, the nuclei were stained using DAPI. Finally, the specimens were visualized and imaged using a fluorescence microscope.

### 4.11. Immunoelectron Microscopy (IEM)

The cells that had been fixed using a specialized fixative for immunoelectron microscopy were subsequently washed three times with PBS. Following this, the cell pellet was then wrapped in agarose. After undergoing dehydration and resin penetration, the cell pellet was embedded in resin and allowed to polymerize at −20 °C for a period of over 48 h. Next, the resin blocks were sliced using an ultramicrotome, and the ultrathin sections were mounted onto formvar-coated nickel grids. After a 30 min blocking process with 1% BSA/TBS at room temperature, the ultrathin section was incubated overnight at a temperature of 4 °C with rabbit polyclonal antibody against beta-conglycinin. Following three washes, the sections were incubated using gold-labeled anti-rabbit IgG (Sigma-Aldrich, St. Louis, MO, USA) for 20 min at room temperature and an additional incubation for 1 h at 37 °C. After undergoing three further washes, the sections were stained using uranium acetate and subsequently dried for examination using an electron microscope.

### 4.12. Impact of Endocytosis Inhibitors and SCG on the Absorption and Transport of Beta-Conglycinin by IPEC-J2 Cells

To assess the role of endocytosis inhibitors on the cellular absorption and transport of beta-conglycinin hydrolysates, the IPEC-J2 cells were seeded in 6-well plates and cultured in DMEM/F12 medium for a duration of 6 days at 37 °C within a CO_2_ incubator. Subsequently, the plates were incubated for 30 min with MβCD at a concentration of 6 mmol/L, CPZ at 7 μg/mL, or amiloride hydrochloride at 50 μM in DMEM/F12 basal medium. Following that, the apical solutions were discarded, and the mixtures containing the aforementioned concentrations of the respective inhibitors, as well as 1.0 mg/mL of beta-conglycinin hydrolysate in DMEM/F12 basal medium, were added and then further incubated in a CO_2_ incubator at 37 °C for 8 h.

Additionally, to explore the effects of SCG on the cellular absorption and transport of beta-conglycinin hydrolysates, the cell monolayers were treated with DMEM/F12 basal medium containing varying concentrations of SCG: 5 mmol/L, 10 mmol/L, and 20 mmol/L. Following a 30 min incubation, the medium was replaced with fresh mixtures containing the aforementioned concentrations of SCG as well as 1.0 mg/mL of beta-conglycinin hydrolysate. The cells were then incubated in a CO_2_ incubator at 37 °C for an additional 8 h.

Following the incubation period, the IPEC-J2 cells were washed six times with ice-cold PBS using a shaker. Subsequently, RIPA lysis buffer containing 1 mmol/L PMSF (Beijing Dingguo Changsheng Biotechnology Co., Ltd., Beijing, China) was added to lyse the cells. The lysed cells were scraped and thoroughly homogenized using a rapid tissue cell grinder (Tianjin Honour Instrument Co., Ltd., Tianjin, China). The intracellular concentration of beta-conglycinin hydrolysate was determined by the competitive ELISA as mentioned above. The absorption rate was calculated by quantifying the proportion of beta-conglycinin hydrolysates that were absorbed by the cells in the presence and absence of the inhibitor or SCG. The suppression rate was calculated by subtracting the absorption rate from 100%.

### 4.13. Statistical Analysis

All data were subjected to statistical analysis by using a one-way ANOVA test in SPSS 26.0 software (SPSS Inc., Chicago, IL, USA). Tukey’s post hoc test was employed to further compare significant differences among the groups. A *p*-value below 0.05 was considered statistically significant.

## 5. Conclusions

The present study demonstrated that the absorption and transport of beta-conglycinin hydrolysates by the IPEC-J2 monolayers occurred through a transcellular pathway, exhibiting both time and quantity dependency. The absorption and transport process was mediated by both clathrin-dependent and caveolae-dependent endocytosis mechanisms. Furthermore, SCG effectively suppressed the absorption and transport of beta-conglycinin hydrolysates by IPEC-J2 monolayers by a quantity-dependent nonlinear model through either clathrin- and caveolae-dependent endocytosis. This research suggested that SCG had the potential to be a promising candidate for suppressing allergen absorption and transport and might act as a preventative agent against allergic responses triggered by soybean protein.

## Data Availability

Data are available upon request from the authors.
